# Overall survival in patients with advanced non-small cell lung cancer with *KRAS G12C* mutation with or without *STK11* and/or *KEAP1* mutations in a real-world setting

**DOI:** 10.1186/s12885-023-10778-6

**Published:** 2023-04-17

**Authors:** Cristina Julian, Navdeep Pal, Anda Gershon, Marie Evangelista, Hans Purkey, Peter Lambert, Zhen Shi, Qing Zhang

**Affiliations:** 1 DNA Way, Genentech, Inc, South San Francisco, CA 94080 USA

**Keywords:** Non-small cell lung cancer, *KRAS G12C*, *STK11*, *KEAP1*, Metastasis, Immunotherapy, Chemotherapy

## Abstract

**Background:**

*KRAS* mutations occur frequently in advanced non-small cell lung cancer (aNSCLC); the *G12C* mutation is the most prevalent. Alterations in *STK11* or *KEAP1* commonly co-occur with *KRAS* mutations in aNSCLC. Using real-world data, we assessed the effect of *KRAS G12C* mutation with or without *STK11* and/or *KEAP1* mutations on overall survival (OS) in patients with aNSCLC receiving cancer immunotherapy (CIT), chemotherapy, or both in first line (1L) and second line (2L).

**Methods:**

Patients diagnosed with aNSCLC between January 2011 and March 2020 in a clinico-genomic database were included. Cox proportional hazards models adjusted for left truncation, baseline demographics and clinical characteristics were used to analyze the effect of *STK11* and/or *KEAP1* co-mutational status on OS in patients with *KRAS* wild-type (WT) or *G12C* mutation.

**Results:**

Of 2715 patients with aNSCLC without other actionable driver mutations, 1344 (49.5%) had *KRAS* WT cancer, and 454 (16.7%) had *KRAS G12C–*positive cancer. At 1L treatment start, significantly more patients with *KRAS G12C*–positive cancer were female, smokers, and had non-squamous histology, a higher prevalence of metastasis and programmed death-ligand 1 positivity than those with *KRAS* WT cancer. Median OS was comparable between patients with *KRAS G12C–*positive and *KRAS* WT cancer when receiving chemotherapy or combination CIT and chemotherapy in the 1L or 2L. Median OS was numerically longer in patients with *KRAS G12C* vs *KRAS* WT cancer treated with 1L CIT (30.2 vs 10.6 months, respectively) or 2L CIT (11.3 vs 7.6 months, respectively). Co-mutation of *STK11* and *KEAP1* was associated with significantly shorter OS in patients receiving any type of 1L therapy, regardless of *KRAS G12C* mutational status.

**Conclusions:**

This real-world study showed that patients with *KRAS G12C–*positive or *KRAS* WT cancer have similar OS in the 1L or 2L when treated with chemotherapy or combination CIT and chemotherapy. In contrast to aNSCLC patients with *EGFR* or *ALK* driver mutations, patients with *KRAS G12C–*positive cancer may benefit from CIT monotherapy. Co-mutation of *STK11* and *KEAP1* was associated with significantly shorter survival, independent of *KRAS G12C* mutational status, reflecting the poor prognosis and high unmet need in this patient population.

## Background

Non-small cell lung cancer (NSCLC) accounts for approximately 85% of all lung cancers [1]. NSCLC is a complex disease consisting of numerous molecular subtypes [2]. Biomarker testing of NSCLC has a direct impact on treatment decisions, and screening tumors for actionable driver mutations is essential before initiating treatment [3]. In general, recommended treatment for patients include targeted therapies for patients with advanced NSCLC (aNSCLC) bearing alterations in driver oncogenes (*EGFR*, *ALK*, *ROS1, BRAF, NTRK, RET,* or *MET*), cancer immunotherapy (CIT) with immune checkpoint inhibitors alone or in combination with chemotherapy [3].

Mutations in *Kirsten Rat Sarcoma Viral Oncogene Homolog* (*KRAS*) are prevalent in NSCLC, occurring in approximately 25 to 40% of patients (≈5–10% in Asian patients) [4–7]. In particular, the *KRAS* glycine 12 to cysteine (*G12C*) activating mutation has the highest prevalence (≈40% of all *KRAS* mutations in NSCLC) [8, 9]. Tumors that harbor *KRAS* mutations are usually among the most aggressive and refractory to treatment [8]. Recently, sotorasib, a mutation-specific inhibitor of KRAS G12C has received marketing authorization in *KRAS G12C*-mutated aNSCLC patients who have received at least one prior systemic therapy [10, 11]. While KRAS G12C inhibitors provide an exciting new second-line or later (2L+) treatment option for patients with *KRAS G12C–*positive tumors, the standard-of-care therapies for first-line (1L) *KRAS G12C*-mutated aNSCLC patients currently remain CIT targeting programmed cell death protein 1 (PD-1) or programmed cell death 1 ligand 1 (PD-L1) alone or combined with chemotherapy.

Patients with tumors that harbor *KRAS* mutations have shown a longer median overall survival (OS) when treated with CIT alone vs chemotherapy alone (28 months (95% CI: 23-NR) vs 11 months (95% CI: 7–25)) [10] or when treated with combination therapy (CIT and chemotherapy) vs chemotherapy alone (21 months (95% CI: 16-NR) vs 14 months (95% CI: 8-NR)) [10]. While patients with *KRAS G12C–*positive cancer may benefit from treatment with CIT alone or in combination with chemotherapy [12, 13], numbers of patients with *KRAS G12C*-positive cancer were low in these previous studies, and it remains unclear how the benefit compares with that seen in patients with *KRAS* wild-type (WT) cancer. Furthermore, somatic genomic alterations in serine/threonine kinase 11 *(STK11)* or kelch like ECH associated protein 1 *(KEAP1)* commonly co-occur with *KRAS* mutations in NSCLC (25–30%) [14], and co-mutation of *KRAS* with *STK11* or *KEAP1* is associated with significantly worse survival [14–16]. It is unknown whether co-occurring mutations affect prognosis and whether differential responses to treatment and consequent effects on survival outcomes exist in these patient populations. This study used real-world data to assess the effect of *KRAS G12C* mutational status on OS in patients with aNSCLC with or without *STK11* and/or *KEAP1* co-mutations who received CIT, chemotherapy, or both in the 1L and 2L using real-world data.

## Methods

### Data source

This study used the nationwide (US-based) deidentified Flatiron Health-Foundation Medicine NSCLC clinico-genomic database (FH-FMI CGDB). Retrospective longitudinal clinical data were derived from electronic health record (EHR) data, comprising patient-level structured and unstructured data, curated via technology-enabled abstraction, and were linked to genomic data derived from FMI comprehensive genomic profiling (CGP) tests in the CGDB by de-identified, deterministic matching [17]. Genomic alterations were identified via CGP of > 300 cancer-related genes on FMI’s next-generation sequencing (NGS) test (FMI sequencing platform[s]: FoundationOne®CDx, FoundationOne®, FoundationOne®Liquid, or FoundationOne®Liquid CDx) [18–20]. Both liquid and solid assays were used in this study. As liquid assays may not detect alterations if shedding of circulating DNA is low, only solid assays were used to define WT *KRAS*, *KEAP1*, and *STK11.*

### Patient population

Eligibility criteria included: (1) aged ≥18 years with aNSCLC newly diagnosed between January 1, 2011, and March 31, 2020; (2) had structured activity within 90 days after aNSCLC diagnosis and had 6 months of follow-up after treatment initiation; (3) did not have functional or likely functional driver alterations (short variants, copy number alterations, or fusions) in *EGFR*, *ALK*, *ROS1*, *BRAF*, *ERBB2*, *MET*, or *RET*; (4) received treatment with CIT (eg, immune checkpoint inhibitors) alone, combination CIT and chemotherapy, or chemotherapy alone and did not receive targeted therapies for driver mutations or nonapproved monotherapy CIT for aNSCLC; (5) displayed no evidence of being diagnosed with other cancers in the database; and (6) had ≥1 definitive (ie, positive or negative) molecular test, including a test for the *KRAS* gene, with results before or after 1L treatment initiation. If a patient had several specimen collections, the closest to initiation of 1L treatment was used. Patients were categorized by *KRAS* mutational status (*G12C* vs WT). The *KRAS* WT group excluded patients with any *KRAS* alteration.

### Outcomes and analysis

The primary outcome was OS in patients with *KRAS* WT or *KRAS G12C–*positive aNSCLC, sorted by the following factors: (1) treatment line (1L or 2L); (2) treatment ([a] CIT alone or in combination with other CIT or with nonchemotherapy; [b] combination CIT and chemotherapy; or [c] chemotherapy alone); 3) presence of mutated *STK11* (m*STK11*) and/or mutated *KEAP1* (m*KEAP1*) vs *STK11* WT and *KEAP1* WT, including any functional status (unknown, likely and known). OS is defined as time from an index date to the date of death for individual patients who have died. Patients without a death date are censored at the last evidence of them being alive, e.g., structured activity in the database.

All statistical analyses were performed with R. Kaplan-Meier (KM) curves, associated medians, and 95% confidence intervals were estimated for survival outcomes. Cox proportional hazards models adjusted for baseline demographics and clinical characteristics (age, sex, race, cancer type [de novo or recurrent], PD-L1 status, any metastasis, tumor mutational burden, histology, and 1L treatment [for 2L analysis only]) were used to analyze the effect of mutational status on OS in patients receiving CIT alone, combination CIT and chemotherapy, or chemotherapy alone. A separate category within a variable was created for the missing values. Adjustments to account for left truncation and immortal bias were applied to the KM analysis and the Cox regression model.

## Results

### Baseline demographics and clinical characteristics

**Fig. 1:**
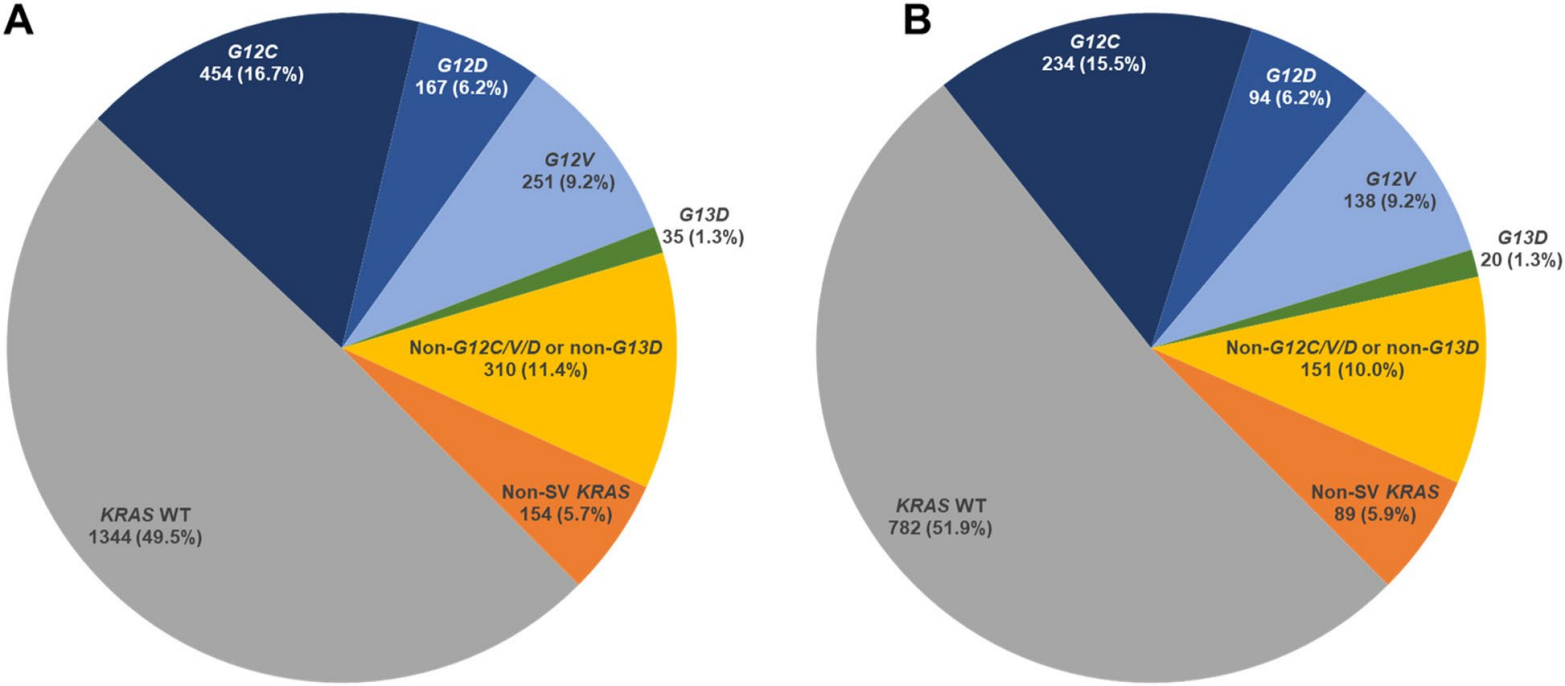
*KRAS* Mutational Status in (**A**) the 1L and (**B**) the 2L Settings. SV, short variant; WT, wild type

**Fig. 2:**
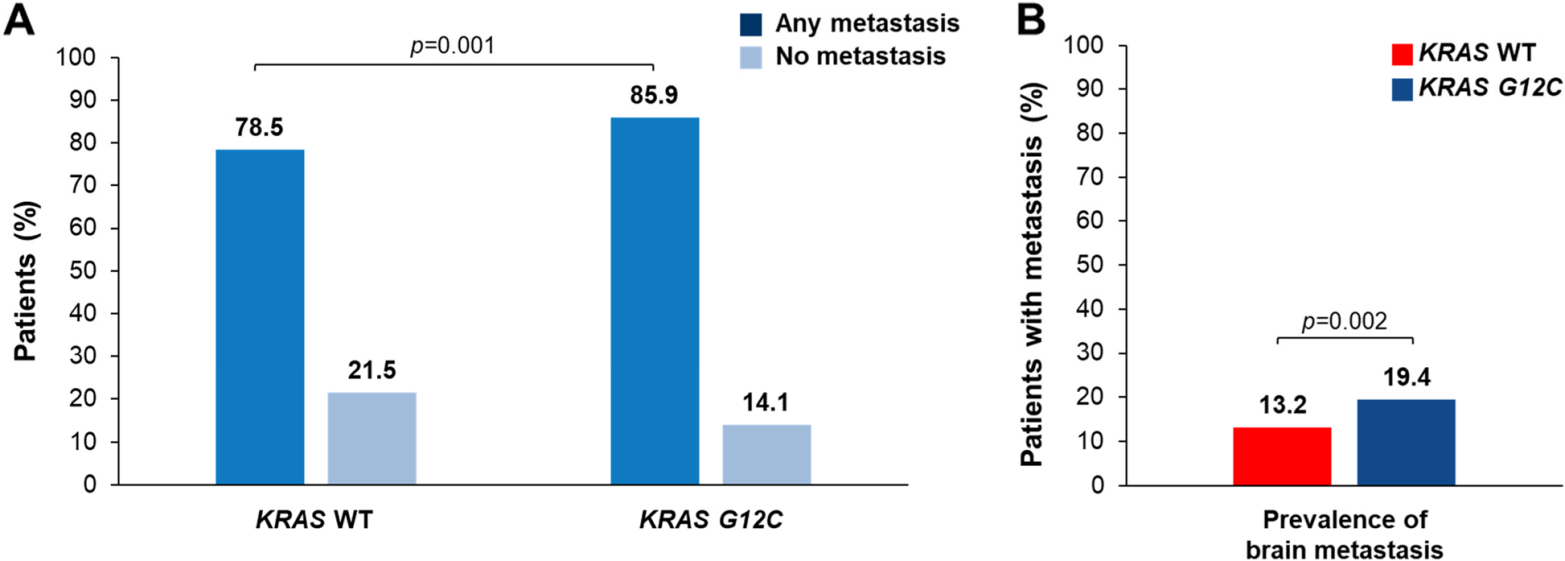
Prevalence of Metastasis in Patients With aNSCLC Treated in the 1L. 1L, first line; aNSCLC, advanced non-small cell lung cancer; WT, wild type

Of the 2715 aNSCLC patients without actionable driver mutations in the 1L setting, 1344 (49.5%) were *KRAS* WT, 454 (16.7%) had tumors with the *KRAS G12C* mutation, 251 (9.2%) had tumors with the *KRAS G12V* mutation, 167 (6.2%) had tumors with the *KRAS G12D* mutation, 35 (1.3%) had tumors with the *KRAS G13D* mutation, 310 (11.4%) had a non-*G12C/D/V* or non-*G13D KRAS* mutation, and 154 (5.7%) had alterations other than short variants such as copy number variations or rearrangements (Fig. 1A). m*STK11-*m*KEAP1* was more prevalent in patients with *KRAS G12C–*positive cancer vs *KRAS* WT cancer (14.3% vs 9.9%; *p =* 0.012) (Table 1). Interestingly, while m*STK11*-*KEAP1* WT was also more prevalent in patients with *KRAS G12C*-positive cancer vs *KRAS* WT cancer (13.0% vs 9.0%; *p* = 0.05), *STK11* WT-m*KEAP1* was more prevalent in patients with *KRAS* WT cancer vs *KRAS G12C*-positive cancer (15.0% vs 7.0%; *p* < 0.001) (Table 1). For 1L treatment, patients with *KRAS* WT cancer received, in order of frequency, chemotherapy alone (69.7%), combination CIT and chemotherapy (19.5%), or CIT alone (10.8%); similarly, the majority of patients with *KRAS G12C–*positive cancer received chemotherapy alone (61.9%), combination CIT and chemotherapy (22.2%), and CIT alone (15.9%) (Table 1). At 1L initiation, patients with *KRAS G12C–*positive cancer had a significantly higher prevalence of metastasis than patients with *KRAS* WT cancer (85.9% vs 78.5%; *p =* 0.001; Fig. 2A). In patients with metastasis, those with *KRAS G12C–*positive cancer had a significantly higher prevalence of brain metastasis than patients with *KRAS* WT cancer (19.4% vs 13.2%; *p =* 0.002; Fig. 2B). In all patients at 1L initiation, 10.4% of patients with *KRAS* WT cancer and 16.7% of patients with *KRAS G12C*-positive cancer had brain metastasis.

**Table 1:** Baseline Characteristics of Patients With aNSCLC by *KRAS* Mutational Status and Treatment Line

	1L			2L		
**Characteristic, n (%)**	***KRAS*** **WT** ***n*** **= 1344**	***KRAS G12C n*** **= 454**	***p***	***KRAS*** **WT** ***n*** **= 782**	***KRAS G12C n*** **= 234**	***p***
**Sex**						
Female	488 (36.3)	281 (61.9)	< 0.001	296 (37.9)	143 (61.1)	< 0.001
Male	856 (63.7)	173 (38.1)		486 (62.1)	91 (38.9)	
**Age at line start**						
18–64 years	479 (35.6)	169 (37.2)	0.572	292 (37.3)	92 (39.3)	0.591
≥ 65 years	865 (64.4)	285 (62.8)		490 (62.6)	142 (60.7)	
**Race**						
White	955 (71.1)	327 (72.0)	0.108	546 (69.8)	167 (71.4)	0.598
Asian	12 (0.9)	≤5 (≤1.1)		9 (1.2)	≤5 (≤2.1)	
Black or African American	107 (8.0)	25 (5.5)		63 (8.1)	17 (7.3)	
Hispanic or Latino		≤5 (≤1.1)				
Other	167 (12.4)	61 (13.4)		113 (14.5)	29 (12.4)	
Missing	103 (7.7)	35 (7.7)		51 (6.5)	≥16 (≥6.8)	
**Smoking status**						
Previous or current	1255 (93.4)	445 (98.0)	< 0.001	727 (93.0)	228 (97.4)	0.018
**Stage at initial diagnosis before progressing to advanced disease**						
Stage I at initial diagnosis	112 (8.3)	52 (11.5)	0.005	65 (8.3)	34 (14.5)	0.001
Stage II at initial diagnosis	80 (6.0)	≥30 (≥6.6)		46 (5.9)	≥7 (≥2.9)	
Stage III at initial diagnosis	326 (24.3)	74 (16.3)		221 (28.3)	41 (17.5)	
Stage IV at initial diagnosis	808 (60.1)	293 (64.5)		440 (56.3)	147 (62.8)	
Unknown stage at initial diagnosis	18 (1.3)	≤5 (≤1.1)		10 (1.3)	≤5 (≤2.1)	
**Histology**						
Non-squamous	745 (55.4)	415 (91.4)	< 0.001	411 (52.6)	214 (91.5)	< 0.001
Squamous	532 (39.6)	19 (4.2)		337 (43.1)	9 (3.8)	
NOS	67 (5.0)	20 (4.4)		34 (4.3)	11 (4.7)	
**TMB**^a^						
High	638 (47.5)	174 (38.3)	< 0.001	171 (21.9)	38 (16.2)	< 0.001
Low	689 (51.3)	235 (51.8)		599 (76.6)	171 (73.1)	
Missing or unknown	17 (1.3)	45 (9.9)		12 (1.5)	25 (10.7)	
**PD-L1**^b^						
High (≥50%)	139 (10.3)	83 (18.3)	< 0.001	73 (9.3)	35 (15.0)	0.003
Low (1–49%)	208 (15.5)	69 (15.2)		116 (14.8)	40 (17.1)	
Negative (< 1%)	228 (17.0)	46 (10.1)		134 (17.1)	21 (9.0)	
Missing or unknown	769 (57.2)	256 (56.4)		459 (58.7)	138 (59.0)	
**Cancer type**						
De novo	809 (60.2)	294 (64.8)	0.095	441 (56.4)	147 (62.8)	0.095
Recurrent	535 (39.8)	160 (35.2)		341 (43.6)	87 (37.2)	
**ECOG PS (**Eastern Cooperative Oncology Group performance status**)**						
0	326 (24.3)	110 (24.2)	0.231	149 (19.1)	49 (20.9)	0.560
1	544 (40.5)	163 (35.9)		371 (47.4)	106 (45.3)	
≥ 2	193 (14.3)	76 (16.8)		143 (18.3)	45 (19.2)	
Missing or unknown	361 (20.5)	105 (23.1)		119 (15.2)	34 (14.5)	
**Drug category**						
Chemotherapy	937 (69.7)	281 (61.9)	0.003	272 (34.8)	83 (35.5)	0.379
CIT	145 (10.8)	72 (15.9)		445 (56.9)	138 (59.0)	
Combination CIT and chemotherapy	262 (19.5)	101 (22.2)		65 (8.3)	13 (5.6)	
***STK11*** **and/or** ***KEAP1*** **mutational status**						
*STK11* WT-*KEAP1* WT	881 (65.6)	240 (52.9)	< 0.001	515 (65.9)	125 (53.4)	0.022
m*STK11*-m*KEAP1*	133 (9.9)	65 (14.3)	0.012	78 (10.0)	31 (13.2)	0.194
m*STK11-KEAP1* WT	129 (9.0)	59 (13.0)	0.05	72 (9.2)	30 (12.8)	0.136
*STK11* WT-m*KEAP1*	201 (15.0)	32 (7.0)	< 0.001	117 (15.0)	19 (8.1)	0.010
**Treatment Start Year Group**						
2009–2015	134 (10.0)	54 (11.9)	0.176	31 (4.0)	15 (6.4)	0.238
2016–2017	377 (28.1)	137 (30.2)		215 (27.5)	73 (31.2)	
2018–2020	833 (62.0)	263 (57.9)		536 (68.5)	146 (62.4)	

*1L* first line; *2L* second line; *aNSCLC* advanced non-small cell lung cancer; *CIT* cancer immunotherapy; *ECOG PS* Eastern Cooperative Oncology Group performance status; *mt* mutation; *NOS* not otherwise specified; *PD-L1* programmed death-ligand 1; *TMB* tumor mutational burden; *WT* wild type

^a^TMB was categorized as high or low using a different threshold (mutations [mut]/megabase[Mb]) for each treatment line; TMB high was defined as ≥10 mut/Mb in the 1L and ≥ 16 mut/Mb in the 2L.

^b^PD-L1high was defined as Tumor Proportion Score ≥ 50%, low 1–49% and negative < 1%

Of the 1745 patients in the 2L setting, 782 (51.9%) were *KRAS* WT, 234 (15.5%) had *KRAS G12C–*positive tumors, 138 (9.2%) had tumors with a *KRAS G12V* mutation, 94 (6.2%) had tumors with a *KRAS G12D* mutation, 20 (1.3%) had tumors with a *KRAS G13D* mutation, 151 (10.0%) had a non-*G12C/D/V* or non-*G13D KRAS* mutation, and 89 (5.9%) had alterations other than short variants (Fig. 1B). *STK11 WT-mKEAP1* and *STK11 WT-KEAP1 WT* were more prevalent in patients with *KRAS* WT cancer, than those with *KRAS G12C-positive* cancer (15.0% vs 8.1%; *p =* 0.01 and 65.9% vs 53.4%, *p =* 0.02, respectively*)* (Table 1). Patients with *KRAS* WT cancer received for 2L treatment, in order of frequency, CIT alone (56.9%), chemotherapy alone (34.8%), and combination CIT and chemotherapy (8.3%); this pattern of results was similar in the 2L setting in patients with *KRAS G12C–*positive cancer (59.0, 35.5, and 5.6%, respectively; Table 1).

In both the 1L and 2L settings, a higher proportion of patients with *KRAS G12C–*positive tumors were female, were former or current smokers, and had tumors with a higher proportion of PD-L1–high and non-squamous histology than patients with *KRAS* WT cancer (Table 1). When limiting to patients with known PD-L1 status, 41.9% (83/198) of *KRAS G12C*-positive patients were PD-L1-high as compare to 24.2% (139/575) of *KRAS* WT patients (*p* < 0.001).

### Effect of *KRAS G12C* mutational status on OS by treatment type

**Fig. 3:**
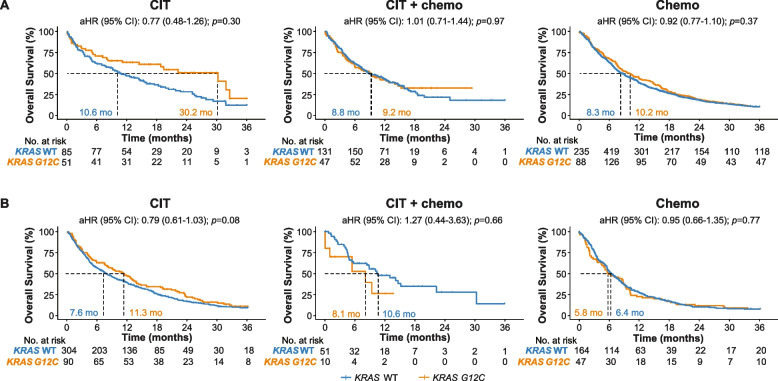
*KRAS* Mutational Status on OS by Treatment in (**A**) the 1L* and (**B**) the 2L^†^. 1L, first line; 2L, second line; aHR, adjusted hazard ratio; CIT, cancer immunotherapy; ns, non-significant; OS, overall survival; PD-L1, programmed cell death 1 ligand 1; TMB, tumor mutational burden; WT, wild type. * *n* = 1798; adjusted by age, sex, race, cancer type, PD-L1, any metastasis, TMB, and histology in the 1L. ^†^ *n* = 1016; adjusted by age, sex, race, cancer type, PD-L1, any metastasis, TMB, histology, and drug category in the 1L

In the 1L setting, patients with *KRAS G12C–*positive cancer treated with CIT alone demonstrated numerical, but not statistically significant, longer OS (median, 30.2 months; 95% CI, 14.5-not reached [NR]) vs patients with *KRAS* WT cancer (median, 10.6 months; 95% CI, 7.9–15.6; adjusted hazard ratio [aHR], 0.77; 95% CI, 0.48–1.26; *p* = 0.30) (Fig. 3A). The median (95% CI) OS in patients with *KRAS* *G12C*-positive vs *KRAS WT* cancer treated with chemotherapy alone was 10.2 (8.0-12.5) months vs 8.3 (7.4-9.4) months (aHR, 0.92; 95% CI, 0.77–1.10; *p* = 0.37). In patients treated with combination CIT and chemotherapy, median OS was 9.2 (5.9-14.0) months in patients with *KRAS* *G12C*-positive tumors vs. 8.8 (7.4-11.9) months in patients with *KRAS* WT tumors (aHR, 1.01; 95% CI, 0.71–1.44; *p* = 0.97) (Fig. 3A).

It is interesting that for patients receiving 1L CIT alone, median OS for patients with *KRAS G12C*-positive cancer is numerically longer than patients with *KRAS* WT cancer. To assess the potential effect of different PD-L1 expression level, we calculated median OS in patients with high (≥50%) PD-L1 expression level, and observed a similar trend of longer OS in patients with *KRAS G12C*-positive cancer treated with CIT alone (median, 32.6 months; 95% CI, 19.4-NR) vs patients with *KRAS* WT cancer (median, 10.0 months; 95% CI, 5.2–17.5; aHR, 0.57; 95% CI, 0.28–1.14, *p* = 0.11) (Table 2).

**Table 2:** OS in patients with *KRAS G12C* or wild-type tumors with high PD-L1 (≥50%) and receiving 1L CIT alone

	N	Death	Median OS (95% CI)	Adjusted* HR (95% CI) ***p***-value
***KRAS*** **wild-type**	65	41	10.0 (5.2–17.5)	Reference
***KRAS G12C***	43	13	32.6 (19.4-NR)	0.57 (0.28–1.14) *p* = 0.11

*Adjusted for age, sex, de novo or recurrent disease, metastasis at baseline, TMB status and histology

It is also interesting that for patients with *KRAS G12C*-positive cancer, median OS for 1L CIT alone is numerically longer than 1L combination CIT and chemotherapy (30.2 months (14.5-NR) vs. 9.2 (5.9–14.0)), we postulate that one key difference in the tumor PD-L1 expression level might at least partially explain this observation. While 1L combination chemotherapy and CIT was approved for patients with NSCLC tumors with any PD-L1 levels, 1L CIT alone was approved only for patients with NSCLC tumors expressing PD-L1. Therefore, patients treated with CIT alone may have tumors with overall higher PD-L1 expression, compared to those treated with the combination chemotherapy and CIT. Indeed, in our study, 59.7% of *KRAS G12C*-positive patients treated with 1L CIT alone vs 17.8% of patients treated with 1L combination chemotherapy and CIT have high PD-L1 expression (Table 3). When limiting to patients with known PD-L1 status, 78.2% of *KRAS G12C*-positive patients treated with 1L CIT alone vs 28.6% of patients treated with 1L combination chemotherapy and CIT have high PD-L1 expression (Table 3). Higher PD-L1 expression might be one reason that the *KRAS G12C*-positive patients receiving 1L CIT alone tend to have longer median OS than patients receiving 1L combination chemotherapy and CIT.

**Table 3:** Percentage of patients with a high (≥50%), low (1–49%) or negative (< 1%) PD-L1 status by treatment type among aNSCLC patients (A) or those with known PD-L1 status (B) receiving 1L CIT alone, combination chemotherapy and CIT, or chemotherapy

**A.**						
***PD-L1***^a^ ***category***	***KRAS G12C***			***KRAS*** **WT**		
	1L CIT alone patients (*N* = 72)	1L combination chemotherapy + CIT patients (*N* = 101)	1L Chemotherapy patients (*N* = 281)	1L CIT alone patients (*N* = 145)	1L combination chemotherapy + CIT patients (*N* = 262)	1L Chemotherapy patients (*N* = 937)
High (≥50%)	43 (59.7)	18 (17.8)	22 (7.8)	65 (44.8)	27 (10.3)	47 (5.0)
Low (1–49%)	10 (13.9)	24 (23.8)	35 (12.5)	23 (15.9)	63 (24.0)	122 (13.0)
Negative (< 1%)	2 (2.8)	21 (20.8)	23 (8.2)	7 (4.8)	74 (28.2)	147 (15.7)
Missing/unknown	17 (23.6)	38 (37.6)	201 (71.5)	50 (34.5)	98 (37.4)	621 (66.3)
**B**.						
***PD-L1***^a^ ***category excluding unknown***	***KRAS G12C***			***KRAS*** **WT**		
	1L CIT alone patients (*N* = 55)	1L combination chemotherapy + CIT patients (*N* = 63)	1L chemotherapy patients (*N* = 80)	1L CIT alone patients (*N* = 95)	1L combination chemotherapy + CIT patients (*N* = 164)	1L chemotherapy patients (*N* = 316)
High (≥50%)	43 (78.2)	18 (28.6)	22 (27.5)	65 (68.4)	27 (16.5)	47 (14.9)
Low (1–49%)	10 (18.2)	24 (38.1)	35 (43.8)	23 (24.2)	63 (38.4)	122 (38.6)
Negative (< 1%)	2 (3.6)	21 (33.3)	23 (28.8)	7 (7.4)	74 (45.1)	147 (46.5)

*1L* first line; *aNSCLC* advanced non-small cell lung cancer; *CIT* cancer immunotherapy; *PD-L1* programmed death-ligand 1; *WT* wild type

^a^PD-L1 high was defined as Tumor Proportion Score ≥ 50%, low 1–49% and negative < 1%

In the 2L setting, median OS in patients with *KRAS G12C–*positive vs *KRAS* WT cancer treated with CIT alone was 11.3 (8.1–13.8) months vs 7.6 (6.3–9.3) months (aHR, 0.79; 95% CI, 0.61–1.03; *p* = 0.08). Median OS in patients treated with chemotherapy alone was 5.8 (4.4–9.3) months vs 6.4 (5.6–7.9) months (aHR, 0.95; 95% CI, 0.66–1.35; *p* = 0.77), and median OS in patients treated with combination CIT and chemotherapy was 8.1 (1.0-NR) months vs 10.6 (8.5-NR) months (aHR, 1.27; 95% CI, 0.44–3.63; *p* = 0.66) (Fig. 3B).

### Effect of *KRAS G12C* mutational status and co-occurring *STK11* and/or *KEAP1* mutations on OS by treatment line

**Fig. 4:**
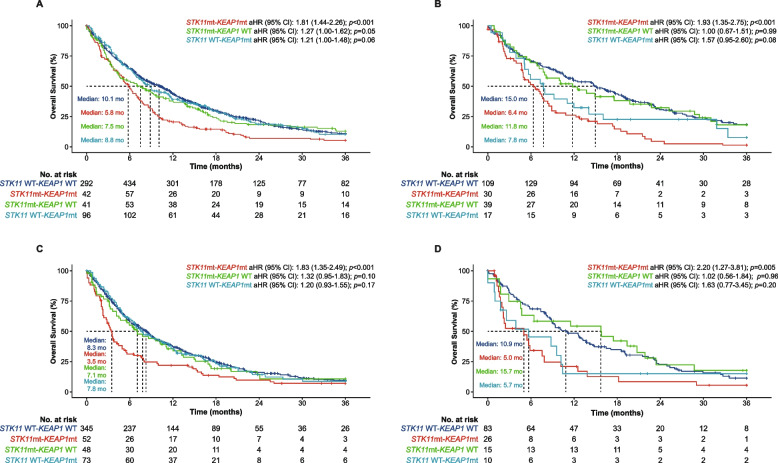
Effect of *STK11* and/or- *KEAP1* Mutations on OS in Patients With aNSCLC in the 1L* With (**A**) *KRAS* WT or (**B**) *KRAS G12C* and in the 2L^†^ With (**C**) *KRAS* WT or (**D**) *KRAS G12C*. 1L, first line; 2L, second line; aHR, adjusted hazard ratio; aNSCLC, advanced non-small cell lung cancer; ns, non-significant; OS, overall survival; PD-L1, programmed cell death 1 ligand 1; TMB, tumor mutational burden; WT, wildtype. * Adjusted by age, sex, race, cancer type, PD-L1, any metastasis, TMB, and histology. ^†^ Adjusted by age, sex, race, cancer type, PD-L1, any metastasis, TMB, histology, and drug category in the 1L. ^‡^ HR comparing *STK11* WT-*KEAP1* WT vs m*STK11* and/or m*KEAP1*

For patients with *KRAS* WT cancer across all treatment types in the 1L setting, significantly shorter median OS was observed in patients with m*STK11*-m*KEAP1* vs *STK11* WT-*KEAP1* WT (5.8 vs 10.1 months; aHR, 1.81; 95% CI, 1.44–2.26; *p <* 0.001) (Fig. 4A). Numerically shorter median OS was also observed in patients with m*STK11-KEAP1* WT vs *STK11* WT-*KEAP1* WT (7.5 vs 10.1 months; aHR, 1.27; 95% CI, 1.00–1.62; *p* = 0.05) and with m*KEAP1*-*STK11* WT vs *STK11* WT-*KEAP1* WT (8.8 vs 10.1 months; aHR, 1.21; 95% CI, 1.00–1.48; *p* = 0.06). A similar pattern of results was observed in patients with *KRAS G12C–*positive cancer: median OS was significantly shorter in patients with m*STK11*-m*KEAP1* vs *STK11* WT-*KEAP1* WT (6.4 vs 15.0 months; aHR, 1.93; 95% CI, 1.35–2.75; *p <* 0.001) (Fig. 4B). Median OS was also numerically shorter in patients with m*KEAP1*-*STK11* WT vs *STK11* WT-*KEAP1* WT (7.8 vs 15.0 months; aHR, 1.57; 95% CI, 0.95–2.60; *p =* 0.08). Patients with m*STK11*-*KEAP1* WT had numerically shorter median OS vs *STK11* WT-*KEAP1* WT, although this difference was not statistically significant (11.8 vs 15.0 months; aHR, 1.00; 95% CI, 0.67–1.51; *p* = 0.99).

For patients with *KRAS* WT cancer across all treatment types in the 2L setting, significantly shorter OS was observed in patients with m*STK11*-m*KEAP1* vs *STK11* WT-*KEAP1* WT (3.5 vs 8.3 months; aHR, 1.83; 95% CI, 1.35–2.49; *p <* 0.001) (Fig. 4C). Similarly, median OS was significantly shorter in patients with *KRAS G12C–*positive cancer with m*STK11*-m*KEAP1* vs *STK11* WT-*KEAP1* WT (5.0 vs 10.9 months; aHR, 2.20; 95% CI, 1.27–3.81; *p* = 0.005) (Fig. 4D). Differences in OS in other mutational groups compared with *STK11* WT-*KEAP1* WT were non-significant.

## Discussion

This retrospective real-world study used an EHR-linked CGDB to assess OS in patients with *KRAS* WT and *KRAS G12C–*positive aNSCLC by treatment line, treatment type, and co-mutations in *STK11* and/or *KEAP1*.

Patients with aNSCLC with *KRAS G12C–*positive tumors were found to have comparable OS relative to patients with *KRAS* WT tumors when receiving combination CIT and chemotherapy or chemotherapy alone in the 1L or 2L; this finding is consistent with majority of the previous literatures using other datasets [12, 13, 21, 22].

Although not statistically significant, potentially due to limited patient counts, patients with *KRAS G12C–*positive cancer treated with 1L CIT showed a trend toward longer survival vs patients with *KRAS* WT cancer. Similar results were observed in patients with high (≥50%) PD-L1 expression level. These results with significantly larger sample size are consistent with previous reports of improved survival with immune checkpoint inhibitors in *KRAS*-mutant aNSCLC [12]. Consistent with the findings of this study, *KRAS* mutations are associated with increased PD-L1 expression in patients with aNSCLC [23] and contribute to immunosuppression [24]. A recent study [25] also found a strong association between mutated *KRAS* and immune biomarkers linked to response to immune checkpoint inhibition [26]. Together these results suggest that patients with aNSCLC whose tumors harbor *KRAS* mutations may have particularly favorable outcomes with CIT [26], which is distinct from *EGFR*-mutated NSCLC [26, 27]. Also, since the single agent KRAS G12C inhibitors appear to have a lower overall response rate compared to the target-specific tyrosine kinase inhibitors [26, 28–30], it is possible that the KRAS G12C inhibitors need to be combined with CIT moving into 1L. Further analyses are needed to evaluate *KRAS* mutational status in patients with aNSCLC treated with CIT alone and to explore the effect of different *KRAS* variants (e.g., *G12D* and *G12V*) on survival relative to *G12C*.

In this study, patients with aNSCLC and concurrent double mutations in *STK11* and *KEAP1* had significantly shorter OS vs patients with *STK11* WT and *KEAP1* WT receiving any type of 1L or 2L therapy regardless of *KRAS G12C* mutational status. This is consistent with recent retrospective analyses conducted using an EHR-linked CGDB that found that mutations in *STK11* and *KEAP1* in aNSCLC were associated with worse outcomes (shorter progression-free survival and OS) in patients treated with anti–PD-L1/PD-1 therapies and platinum-based chemotherapy [31]. Our results build on these previous findings and further underscore the poor prognosis and high unmet medical need that exists in patients with aNSCLC with co-occurring mutations in *STK11* and *KEAP1,* with either *KRAS* WT cancer or *KRAS G12C–*positive cancer. If *KRAS G12C*-positive patients with *STK11* co-mutations are sensitive to the KRAS G12C inhibitors, this may serve as the rationale of expediting the exploration of single-agent KRAS G12C inhibitors in certain biomarker-selected patient sub-population in 1L.

Initial studies of KRAS G12C inhibitors excluded patients with active brain metastasis [11]. Our study showed that *KRAS G12C*-positive patients have a higher prevalence of brain metastasis as compared to patients with *KRAS* WT tumors. It is therefore critical to evaluate whether KRAS G12C inhibitors can be beneficial for patients with brain metastasis and brain-penetration may be a key consideration for future generations of KRAS G12C inhibitors.

This study has several limitations. CGDB data are generated from real-world clinical practice; thus, some data may have been miscoded or may be subject to errors encountered in an oncology clinic. The data do not capture information about patients’ history or treatment outside of the specific cancer care site, which may lead to underreporting or missing data. Limited data exist from patients attending or beginning treatment elsewhere, such as academic medical centers, as the CGDB largely reflects community oncology treatment information. The study population was comprised of patients who received treatment in the United States, and the results may not be generalizable to patients treated globally. For specimen collection, patients were categorized as having *KRAS G12C* or *KRAS* WT cancer based on the closest specimen collection to index date (1L or 2L treatment initiation). With this approach, patients may have developed a *KRAS* mutation after receiving therapy; however, sensitivity analyses were conducted to evaluate selection bias. Analyses were rerun in patients with a specimen collection date within 90 days of the index date (1L or 2L treatment initiation), and results were found to be consistent. We categorized patients with *KRAS G12C*, *KEAP1,* and *STK11* mutational status using both solid and liquid assays; patients with cancer that was categorized as WT were assigned based on solid assays only. To avoid selection bias, we further restricted and subsequently reperformed our analyses of patients categorized using solid assays only. The results remained consistent; however, to ensure a larger sample size, patients whose mutational status was determined using liquid assays were included in the final analysis. Due to the entry selection rules, the CGDB is inherently left truncated. For inclusion in the cohort, patients were required to have undergone NGS testing by FMI, and, therefore, must have been alive until the date of the NGS test. The analyses were adjusted for left truncation to control for this potential immortal bias. Eastern Cooperative Oncology Group performance statuses were missing in 14 to 23% of patients and not adjusted for due to this high missingness. A higher proportion of patients with *KRAS G12C–*positive tumors had non-squamous histology than patients with *KRAS* WT tumors. Although squamous, NOS vs non-squamous histology was adjusted for, no further details such as adenocarcinoma was available in the CGDB. This study included patients with locally advanced (stage IIIB and IIIC) and metastatic (stage IV) diseases, which could have also progressed from an initial diagnosis at early stages (stage I, II and IIIA). Although stage at initial diagnosis was not adjusted for, cancer type (de novo vs recurrent) and presence of metastasis at baseline were included in the model to adjust for heterogeneity in patient population. Finally, this study included a high percentage of patients with missing PD-L1 data; PD-L1 status was unknown for > 50% of patients in each group. Despite these limitations, these findings highlight the importance of evaluating genomic alterations in clinical practice to better understand how selection of treatment and therapy type affect survival in patients with tumors bearing genomic alterations. This study offers a unique design advantage to the published literature to date. Other studies have assessed the effect of *STK11* and/or *KEAP1* mutations either in patients whose tumors harbor *KRAS* mutations only, or in a mixed patient population whose tumors could harbor either *KRAS* WT or *KRAS* mutations [32–35]. This study evaluated survival benefit with different 1L and 2L therapies in patients with *KRAS G12C* or WT cancer with or without *STK11* and/or *KEAP1* mutations, providing additional insights into these frequently co-occurring mutations. Further analyses with a larger sample size evaluating the interplay between *KRAS* and *STK11* and/or *KEAP1* by select treatment types are warranted.

Results from this study may inform personalized treatment for patients with *KRAS*-mutated NSCLC, as certain combinations of mutations in *KRAS* and other genes may generate biological diversity that may respond to tailored treatment [36]. Together, these results may enable personalized care and help optimize patient outcomes.

## Conclusions

Patients with aNSCLC with *KRAS G12C* mutations who were treated with 1L and 2L chemotherapy, and combination CIT and chemotherapy had similar OS compared with patients with *KRAS* WT cancer. Patients with *KRAS G12C–*positive cancer showed a (non-significant) numerically longer OS when treated with CIT monotherapy. Further validation with independent datasets is warranted. Co-occurring mutations in *STK11* and *KEAP1* were associated with significantly shorter OS in the 1L and 2L settings, regardless of *KRAS G12C* or WT status. This study highlights the importance of evaluating genomic alterations in clinical practice to better understand the interplay between treatment type and survival.

## Data Availability

The data that support the findings of this study originated from Flatiron Health, Inc., and Foundation Medicine, Inc. These deidentified data may be made available upon request and are subject to a license agreement with Flatiron Health and Foundation Medicine, Inc.; interested researchers should contact Flatiron Health at n to determine licensing terms.

## Abbreviations

1L: First line
2L: Second line
aHR: Adjusted hazard ratio
aNSCLC: Advanced non-small cell lung cancer
CGBD: Clinico-genomic database
CGP: Comprehensive genomic profiling
CIT: Cancer immunotherapy
EHR: Electronic health record
FH-FMI: Flatiron Health-Foundation Medicine
IRB: Institutional Review Board
*KEAP1*: kelch like ECH associated protein 1
KM: Kaplan-Meier
*KRAS*: *Kirsten Rat Sarcoma Viral Oncogene Homolog*
NGS: Next-generation sequencing
NOS: Not otherwise specified
ns: Non-significant
NR: Not reached
NSCLC: Non-small cell lung cancer
OS: Overall survival
*p*: *p*-value
PD-1: Programmed cell death protein 1
PD-L1: Programmed cell death 1 ligand 1
*STK11*: Serine/threonine kinase 11
SV: Short variant
TMB: Tumor mutational burden
WT: Wild type
